# A Rare Case of Chickenpox Infection Complicated by Hip Septic Arthritis

**DOI:** 10.7759/cureus.45930

**Published:** 2023-09-25

**Authors:** Khadeejeh Alfroukh, Mosa R Abu Sabha, Zinah A Bairmani, Mohammed G Tomizi, Abdelwadod A Abuturki

**Affiliations:** 1 Department of Internal Medicine, Al-Ahli Hospital, Hebron, PSE; 2 Department of Internal Medicine, Faculty of Medicine, Al Quds University, Jerusalem, PSE; 3 Department of Pharmacology & Experimental Therapeutics, Thomas Jefferson University, Philadelphia, USA

**Keywords:** musculoskeletal complications, complication, immuno-competent, septic arthritis of hip, varicella zoster (chicken pox)

## Abstract

Chickenpox represents a viral malady characterized by the emergence of vesicular skin eruptions. This ailment is frequently encountered during childhood and typically manifests a benign course devoid of complications. Among the prevalent complications, secondary bacterial skin infections ranging from superficial impetigo to subcutaneous abscesses are most frequently observed. Instances of musculoskeletal complications, such as septic arthritis and osteomyelitis, are rarely observed. In any patient presenting complaints of bone pain or arthralgia, either during varicella eruptions or during the healing process, it is imperative to maintain a vigilant consideration for the potential manifestation of septic arthritis and osteomyelitis. Timely diagnosis holds paramount importance, as the administration of appropriate antibiotics can effectively forestall the necessity for surgical interventions and mitigate the risk of sequela. In this context, we present a case wherein chickenpox resulted in the complication of right hip septic arthritis.

## Introduction

Chickenpox, a prevalent viral affliction during childhood, is characterized by the emergence of vesiculobullous eruptions and generally pursues a benign clinical course [1]. While serious complications are well-documented in immunocompromised individuals, particularly those harboring T-cell deficiencies and tumors, such occurrences remain rare in patients with intact immune function [2].

Among the array of complications, the foremost are secondary bacterial skin infections encompassing a spectrum from superficial impetigo to subcutaneous abscesses [3]. Additionally, relatively common complications include encephalitis, cerebellar ataxia, pneumonia, hepatitis, and thrombocytopenia [4].

Although musculoskeletal complications such as septic arthritis and osteomyelitis have been recognized as potential sequelae of chickenpox, their occurrence remains infrequent [5]. Septic arthritis of the hip stands as a bona fide orthopedic urgency, where delayed diagnosis or intervention may culminate in irreparable joint damage. Notably, *Staphylococcus aureus* serves as the prevalent causative microorganism, accounting for 50% of cases [6]. Early and precise diagnosis plays a pivotal role in averting complications like joint deterioration, the spread of infection resulting in osteomyelitis, or the development of nerve lesions [7]. Consequently, the significance of early diagnosis and efficacious management cannot be overstated. Herein, we report an unusual case of chickenpox complicated by right hip septic arthritis in a 15-year-old male patient.

## Case presentation

**Figure 1:**
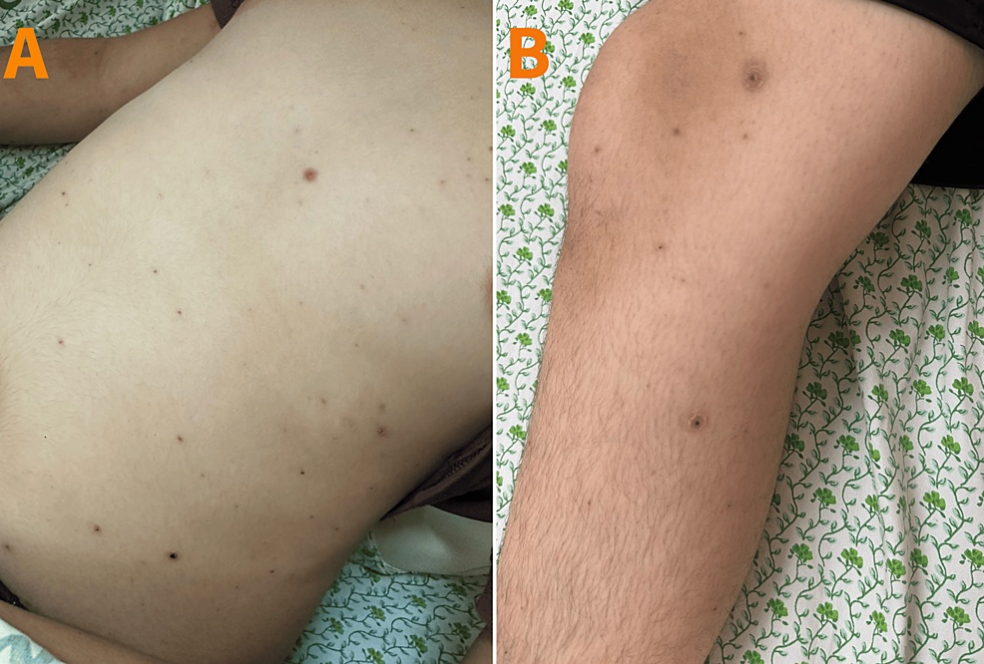
Panels A and B show crusted skin lesions consistent with chickenpox

A 15-year-old male patient, diagnosed with chickenpox, presented on the tenth day of cutaneous vesicular eruption at the emergency department (ED) complaining of worsening pain in the right hip for eight days. He rated the pain as 9/10, characterized as dull to sharp, localized anteriorly on the right hip, radiating to the lower leg, and aggravated by minimal leg flexion and internal rotation. Accompanying the pain was subjective chills. Upon ED assessment, the patient was in mild distress due to the pain, showing systemic wellness but with a fever (oral temperature of 39.21°C). Tenderness was noted upon palpation of the right hip without observable erythema. There was also a notably restricted range of motion in the right leg, and crusted skin lesions were evident without signs of superinfection (Figures 1A-1B). Consequently, due to the suspicion of septic arthritis, the patient was admitted to the medical ward, and laboratory results upon admission are provided in Table 1. The elevated C-reactive protein, erythrocyte sedimentation rate, and procalcitonin levels suggest a bacterial infection with an inflammatory response. 

**Table 1:** The initial laboratory tests

Lab Test	Result	Reference ranges
Hemoglobin	11.3	14-18 g/dl
Mean corpuscular volume	73	82-94 fL
Hematocrit	36.7	40-52%
Mean corpuscular hemoglobin	22.5	27-31pg
White blood cells	8500	5000-10000/mm^3^
Neutrophils	2200	2500-2700/ul
Lymphocyte s	380	1500-3500/ul
Platelet s	535000	150000-400000/mm^3^
Erythrocyte sedimentation rate	60	0-15 mm/h
C-reactive protein	201	Up to 6 mg/L
Procalcitonin	2.31	>=0.5ng/ml presence of bacterial infection
Ferritin	1132	Males, 21.8-275 ng/ml; children, 7-140 ng/ml

**Figure 2:**
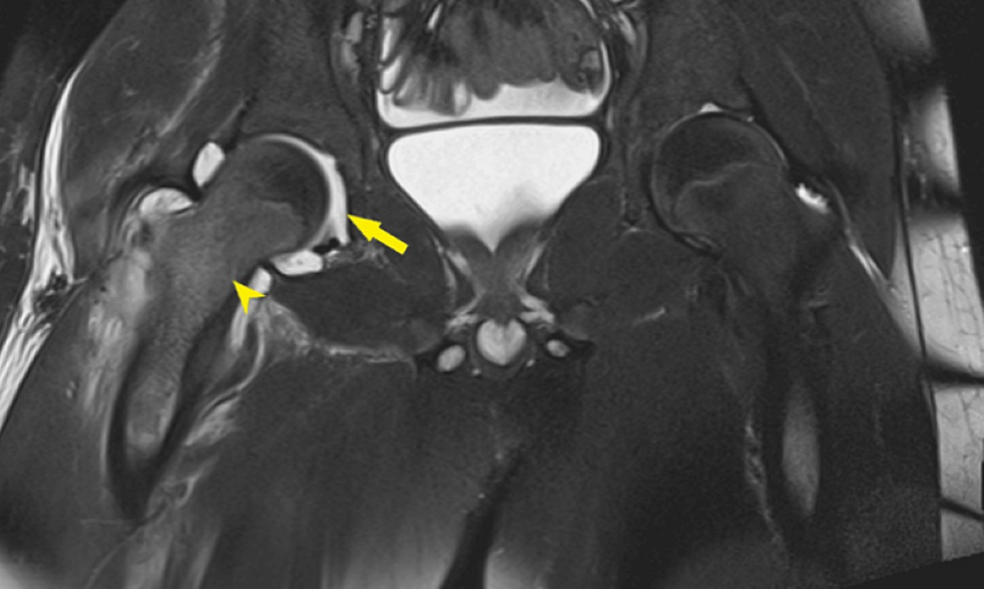
T2-weighted MRI image in the coronal plane shows mild right hip joint effusion (arrow)and bone marrow edema of the femoral neck and proximal shaft (arrowhead)

**Figure 3:**
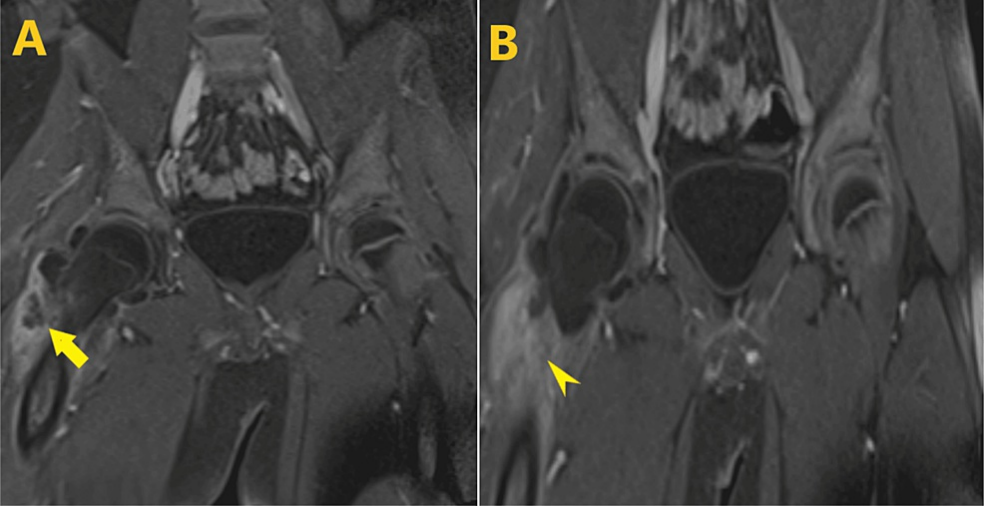
(A) T1-weighted enhanced MR images in the coronal plane showing thin enhancement of the synovium (arrow); (B) associated with surrounding soft tissue edema and enhancement (arrowhead)

Subsequent magnetic resonance imaging with contrast indicated findings suggestive of severe septic arthritis in the right hip, accompanied by a prominent inflammatory process involving the surrounding soft tissue. Minimal pelvic free fluid and bilateral small lymph nodes were also noted (Figures 2-3). Initially, the patient was administered empiric intravenous vancomycin, ceftazidime, and doxycycline.

A rheumatology consultation was conducted to rule out underlying rheumatologic disorders. All immunological markers, including antinuclear antibodies (ANA), rheumatoid factor (RF), anti-cyclic citrullinated peptide (anti-CCP), perinuclear antineutrophil cytoplasmic antibody (P-ANCA) or cytoplasmic ANCA (C-ANCA), returned negative.

The orthopedics team evaluated the patient and performed a joint aspiration procedure under interventional radiology. During the procedure, irrigation with 5 mL of normal saline was carried out in the joint space and anterior soft tissues, and the irrigated fluid was sent for culture and sensitivity testing. The results of the joint aspirate analysis confirmed the diagnosis of septic arthritis Table 2.

**Table 2:** Synovial fluid analysis PMNs: Polymorphonuclear neutrophils

Joint fluid	Clarity	Appearance	White blood cell count /mm3	PMNs%	Gram stain	Crystals
Joint aspirate	Opaque	Yellow	95000	90%	Positive	Negative
Reference (normal)	Transparent	Clear	<200	<25%	Negative	Negative

Broad-spectrum empiric antibiotics were reintroduced and later narrowed down to IV cefazolin after *Staphylococcus aureus* was identified in aerobic cultures from the joint aspirate and the blood culture without any specific antibiotic resistance identified.

The patient's condition improved significantly, with intravenous antibiotics administered for a duration of seven days. Subsequently, he was discharged to continue oral antibiotic treatment for an additional three weeks. Although a follow-up appointment was scheduled, the patient did not return for further evaluation. This case underscores the rare occurrence of serious complications of varicella-zoster virus in immunocompetent patients, including musculoskeletal complications such as septic arthritis.

## Discussion

We present an unusual case of a 15-year-old immunocompetent male who was diagnosed with chickenpox and initiated on acyclovir after developing the characteristic rash. Ten days later, he presented to the emergency department with severe right hip pain, fever, and limited mobility. Due to suspicions of septic arthritis, he was admitted to the hospital. Subsequent investigations unveiled heightened inflammatory indicators, joint fluid accumulation, and MRI findings that suggested the condition. This case highlights the unusual complication of septic arthritis in an immunocompetent patient with varicella infection.

Varicella, also known as chickenpox, is a contagious viral infection caused by the varicella-zoster virus, a Herpesvirus. It is characterized by a prodromal period of flu-like symptoms followed by an intense pruritic vesicular rash that occurs in crops at different stages of healing [8].

The most common bacterial superinfection is cellulitis, while less frequent complications include encephalitis, pneumonia, and disseminated infection. Adults may experience more severe infections compared to children and are at greater risk of pneumonia [4]. Musculoskeletal complications like osteomyelitis, septic arthritis, and pyomyositis are rare, occurring at an estimated rate of one in every 10,000 varicella cases [5].

Although septic arthritis is less common than osteomyelitis, it can lead to a destructive inflammatory process in the joint, resulting in significant morbidity and mortality [9]. *Staphylococcus aureus* is one of the major organisms implicated in post-varicella musculoskeletal complications [10]. The pathogenesis of septic arthritis in varicella infection is not fully understood but may include the direct extension of bacteria from the compromised skin barrier of varicella lesions, dissemination from secondarily infected varicella skin lesions, or previous aseptic effusion that becomes infected [11].

This case holds notable significance for several reasons. Firstly, there is limited available data on hip septic arthritis as a complication of varicella infection. Additionally, approximately 50% of hip septic arthritis cases occur in individuals younger than two years old [12]. Moreover, this case underscores the necessity of considering septic arthritis as a potential diagnosis for limb pain following varicella infection. Clinical suspicion should remain high, as arthralgia or aseptic arthritis, while more common, may overshadow the possibility of septic arthritis. It's important to note that both aseptic arthritis and arthralgia typically exhibit migratory symptoms and resolve within three days [13].

## Conclusions

This case serves as a reminder that septic arthritis should be considered as a potential complication in cases of arthritis associated with varicella, even in immunocompetent individuals. It underscores the importance of early diagnosis, including the utilization of MRI and arthrocentesis, as well as the timely initiation of appropriate management to prevent the development of severe complications associated with septic arthritis.
